# Time-series prediction and detection of potential pathogens in bloodstream infection using mcfDNA sequencing

**DOI:** 10.3389/fcimb.2023.1144625

**Published:** 2023-05-11

**Authors:** Yinghao Cao, Tingting Jiang, Yanfeng Lin, Xiaofeng Fang, Peipei Ding, Hongbin Song, Peng Li, Yanjun Li

**Affiliations:** ^1^ Department of Clinical Laboratory Medicine, School of Medicine, South China University of Technology, Guangzhou, China; ^2^ Department of Clinical Laboratory Medicine, The Sixth Medical Center of People's Liberation Army (PLA) General Hospital of Beijing, Beijing, China; ^3^ Department of Epidemiology and Biostatistics, School of Public Health, An Hui Medical University, Hefei, China; ^4^ Biosecurity Department, Chinese People's Liberation Army (PLA) Center for Disease Control and Prevention, Beijing, China; ^5^ School of Laboratory Medicine and Biotechnology, Southern Medical University, Guangzhou, China

**Keywords:** microbial cell free DNA, next-generation sequencing, blood stream infection, pathogens, microbiology

## Abstract

**Introduction:**

Next-generation sequencing of microbial cell free DNA (mcfDNA-seq) has emerged as a promising diagnostic method for blood stream infection (BSI) and offers the potential to detect pathogens before blood culture. However, its application is limited by a lack of clinical validation.

**Methods:**

We conducted sequential mcfDNA-seq on blood samples from ICU participants at high risk of BSI due to pneumonia, or intravascular catheterization; and explored whether mcfDNA-seq could diagnose and detect pathogens in advance of blood culture positivity. Blood culture results were used as evaluation criteria.

**Results:**

A total of 111 blood samples were collected during the seven days preceding and on the day of onset of 16 BSI episodes from 13 participants. The diagnostic and total predictive sensitivity of mcfDNA-seq were 90% and 87.5%, respectively. The proportion of pathogenic bacteria was relatively high in terms of both diagnosis and prediction. The reads per million of etiologic agents trended upwards in the days approaching the onset of BSI.

**Discussion:**

Our work found that mcfDNA-seq has high diagnostic sensitivity and could be used to identify pathogens before the onset of BSI, which could help expand the clinical application of mcfDNA-seq.

## 1 Introduction

Infectious diseases remain among the major threats to humanity (Casadevall, 2017). Bloodstream infection (BSI) is one of the most lethal infections. Undetermined or delayed etiologic diagnoses often result in inadequate treatment, prolonged hospitalizations, readmissions, and increased mortality and morbidity (Ewig et al., 2002; Bleeker-Rovers et al., 2007). Furthermore, BSI mortality rates rise with delays of appropriate antibiotic therapy (Lodise et al., 2007; Zimlichman et al., 2013; Sterling et al., 2015). Rapid etiologic diagnosis and the prompt initiation of pathogen-directed therapy are crucial to improving clinical outcomes (Evans et al., 2021).

Blood culture is currently regarded as the gold standard for diagnosing BSI. However, blood culture sensitivity relies to a large degree on sample volume, requiring 40-80 mL blood to diagnose the majority of BSI, which is extremely damaging to the participant (Cockerill et al., 2004; Riedel et al., 2008). Although a predictive test to enable preemptive, pathogen-directed therapy could reduce morbidity and mortality, no such reliable method has been developed. In this context, the development of a blood-sparing and predictive etiologic diagnostic test is an unmet medical need.

Plasma microbial cell-free DNA sequencing (mcfDNA-seq) has emerged as a diagnostic tool and predictive test that may meet such a need. McfDNA-seq could detect a wide range of pathogens. The first diagnostic assay using mcfDNA-seq identified pathogens in lung transplant recipients in 2015 (De Vlaminck et al., 2015). Subsequent studies have reported that plasma mcfDNA-seq may identify the etiologic agents of BSI (Grumaz et al., 2016; Long et al., 2016; Jing et al., 2021; Wang et al., 2021).

Moreover, increased plasma cfDNA may indicate the onset of sepsis (Martins et al., 2000; Rhodes et al., 2006; Grumaz et al., 2016). The incidence rates of hospital-acquired BSI were 75% and 25% before and after transfer to ICU, respectively (Tabah et al., 2012; Timsit et al., 2020). ICU-acquired BSI was caused by catheter-related infections (21%), nosocomial pneumonia (21%), intra-abdominal infections (12%) or no definite source (24%) (Tabah et al., 2012). BSI may be predictive and mcfDNA-seq has the potential to predict BSI. However, its possible clinical role of BSI prediction has rarely been explored (Goggin et al., 2020).

In this study, we conducted sequential mcfDNA-seq on blood samples from participants hospitalized in ICU and at high risk of BSI, with the goal of evaluating the diagnostic performance of mcfDNA-seq and exploring whether it could identify etiologic agents before BSI develops.

## 2 Materials and methods

### 2.1 Participants and ethics

**Figure 1:**
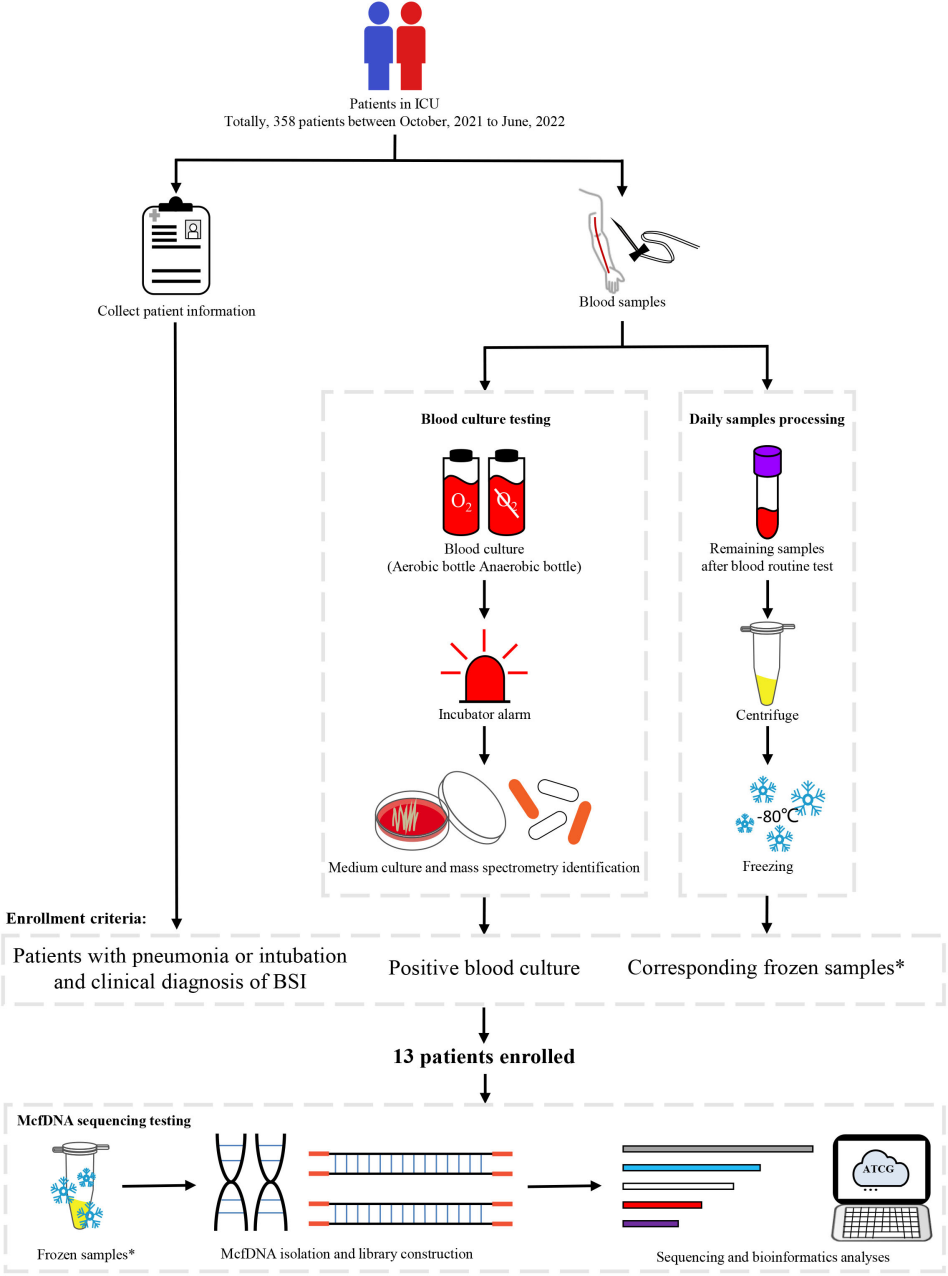
Study workflow. *Residual samples were used for mcfDNA-seq after participants were enrolled. Sequencing samples needed to be obtained during three or more days within the seven days prior to the collection time of the positive blood culture.

Participants in ICU receiving treatment were enrolled from October 2021 to June 2022 at the sixth medical center of PLAGH. The enrollment criteria were (1) participants with high risks for BSI (with pneumonia or receiving intravascular catheterization); (2) participants getting definitive diagnosis of BSI and having blood culture as a criterion; (3) mcfDNA-seq samples available for three or more days within the seven days prior to the time of collection of the positive blood culture. In total, we enrolled 13 participants for our research (**Figure 1**). Information of participants were collected from the electronic medical record. This study was approved by the ethics committee of the sixth medical center of PLAGH (Ethical acceptance number: H2KY2022-41). Informed consent was not required because no additional processes were done to participants and participant information was anonymized.

### 2.2 Specimen collection and processing

Blood samples were collected using standard aseptic technique and used for blood culture and mcfDNA-seq. Samples that were injected into blood culture bottles were processed in BacT/ALERT^®^ 3D (bioMerieux, Inc.). Blood from positive-alarm culture bottles was extracted and inoculated into medium and incubated at 35°C for more than 24h in MCO-15AC (SANYO). Species-level identification was performed using Microflex LH/SH(Bruker).

Residual blood specimens obtained after blood routine tests were subjected to mcfDNA-seq. Only samples that otherwise would have been discarded were used; no samples were collected specifically for this study. After blood routine tests, approximately 500 to 600μL plasma would remain. The residual blood samples were aspirated without mixing and centrifuged at 1600g for 10mins at 4°C, and the supernatant would be centrifuged again at 16000g for 10 min at 4°C. Finally. 350μL supernatants containing plasma and cell-free DNA were then transferred into a new tube and frozen at -80°C for further experiments.

### 2.3 mcfDNA sequencing and bioinformatic analysis

500μL sample (350μL supernatants plus 150μL ultraclean water) was taken for nucleic acid extraction using QIAamp MinElute ccfDNA Mini Kit (50) according to the manufacture’s instruction. The extracted mcfDNA were quantified by Qubit 3.0 fluorometer with Qubit dsDNA HS Assay Kit (ThermoFisher). The initial amount of each sample was 2.5ng. DNA libraries were constructed using MGIEasy Cell-free DNA Library Prep Kit. The constructed libraries were qualified using Qsep100 (Guangding Biotechnology, Taiwan, China). Sequencing was performed on MGISEQ-2000 with PE100 strategy. The raw reads were aligned using Centrifuge1.0.4 with the p+h+v database and the value of min-hitlen was set as 30 (Kim et al., 2016). The p+h+v database which consists of prokaryotic, human and viral genomes helped to obtain the reads of microorganism. In the data generated by Centrifuge 1.0.4, some reads which were aligned to specific species also were aligned to a group or complex. Only the reads aligned to a specific species represent reads that belong to a specific species. For estimating sensitivity, quantitative results of detected organisms were documented as positive or negative. Samples with pathogens identified by mcfDNA-seq (if mcfDNA-seq and blood culture results were concordant) were considered positive. For blood cultures yielding multiple isolates, samples with all of the isolates identified by mcfDNA-seq were considered positive.

### 2.4 Data definitions

The time of collection of a positive blood culture was defined as the onset of a BSI episode. Samples collected on the day of BSI onset were defined as the diagnostic sample and used to evaluate the performance of mcfDNA-seq, while samples collected during the seven days prior to the onset of BSI were defined as predictive samples and used to explored whether mcfDNA-seq could identify etiologic agents in advance.

Daily detective sensitivity was defined as the proportion of positive sequencing samples in all sequencing samples on a same day. Depending on the sampling time, daily detective sensitivity could be divided into diagnostic sensitivity estimated from samples collected on onset of BSI and daily predictive sensitivity estimated from samples collected prior to onset of BSI. Total predictive sensitivity was defined as the proportion of BSIs in which pre-onset mcfDNA-seq identified the same pathogen as blood culture on onset (Goggin et al., 2020).

For inter-sample comparison, reads per million (RPM) was used to normalize the effect of different numbers of raw reads as described in a previous study (Gu et al., 2021). RPM was defined as the number of species reads divided by the number of raw reads of a sample, and then multiplied by one million.

## 3 Results

### 3.1 Participant characteristics and sample distribution

**Figure 2:**
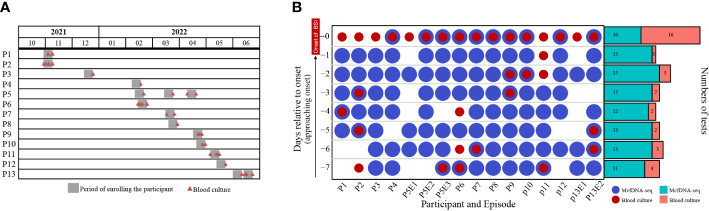
**(A)** The timeline of participant enrollment and **(B)** sample distribution. P and E indicate participant and episode, respectively. For example, P5E1 indicates the first BSI of Participant 5. Day -7 indicates the seventh day before the onset of BSI, while Day 0 indicates the day of BSI diagnosis.

Between October 2021 and June 2022, a total of 3099 blood samples from 358 participants in ICU were obtained during daily collections for clinical laboratory testing. Thirteen participants met our inclusion criteria (**Table 1**). The median participant age was 76 years (range: 32-94 years) and 84.6% (11 participants) were male. Ten participants had both pneumonia and intravascular catheterization, while two participants were diagnosed with pneumonia but did not have vascular catheters, and one participant had received intravascular catheter but had no other BSI risk factor. Seven participants had received antibiotic therapy for at least one day during the week before BSI onset. Participants were enrolled at a nearly constant rate throughout the research period (**Figure 2A**). Sixteen BSI episodes occurred among the thirteen participants. Except for participants P5 and P13, who had developed three and two BSI episodes, respectively, other participants experienced only one BSI episode.

**Table 1:** Participant characteristics.

Characteristic	Number
Total participants	13
Age	76 (32-94)
Sex	
Male	11
Female	2
Risk factors*	
Pneumonia	12
Intravascular catheterization	11
Empiric antibiotic therapy	7
Mortality^#^	6

*Getting more than seven days prior to positive blood culture.

^#^Deaths due to septic shock.

The sample distribution by day was shown in **Figure 2B**. A total of 111 samples were collected. However, samples were not obtained every day from every participant. Twenty-two samples were used for both blood culture and mcfDNA-seq, while 11 samples were used only for blood culture and 78 samples were subjected only to mcfDNA-seq (**Figure 2B**). All participants had positive blood cultures on the day of BSI onset (BSI case definition), while blood cultures were obtained from only nine participants before BSI onset. An average of 6.9 (range: 4-8) samples were collected per BSI episode and an average of 6.25 (range: 3-8) samples were subjected to mcfDNA-seq.

### 3.2 Pathogens identified by blood culture

**Figure 3:**
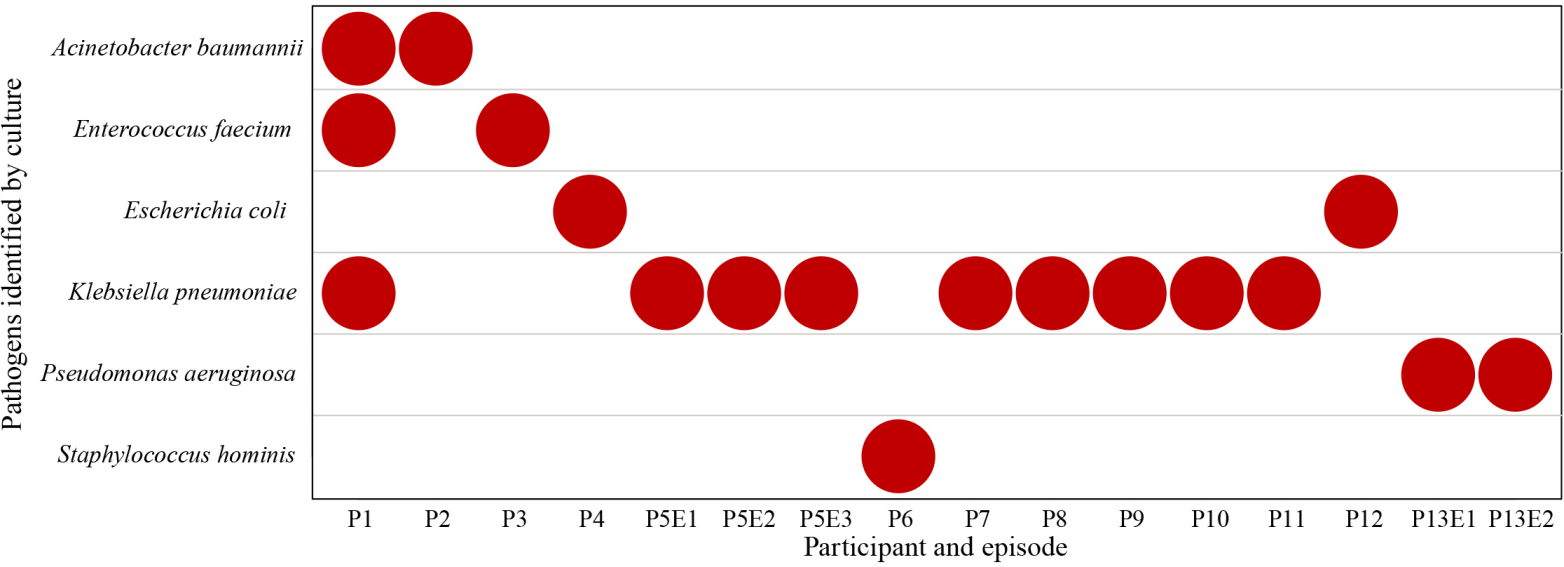
Pathogens identified at onset of BSI by culture.

Thirty-three samples were cultured, of which seventeen obtained prior to the onset of BSI from nine participants were negative and the sixteen samples obtained on the onset of BSI were positive. Six different pathogens were identified, including *Klebsiella pneumoniae* (9/16), *Acinetobacter baumannii* (2/16), *Enterococcus faecium* (2/16), *Escherichia coli* (2/16), *Pseudomonas aeruginosa* (2/16) and *Staphylococcus hominis* (1/16) (**Figure 3**). Participant P1 was co-infected with *A. baumannii*, *E. faecium* and *K. pneumoniae*, while other BSIs were monobacterial. Participants P5 and P13 had recurrent BSI episodes caused by the same pathogen, *K. pneumoniae* and *P. aeruginosa*, respectively.

### 3.3 Diagnostic and predictive performance of mcfDNA-seq

**Figure 4:**
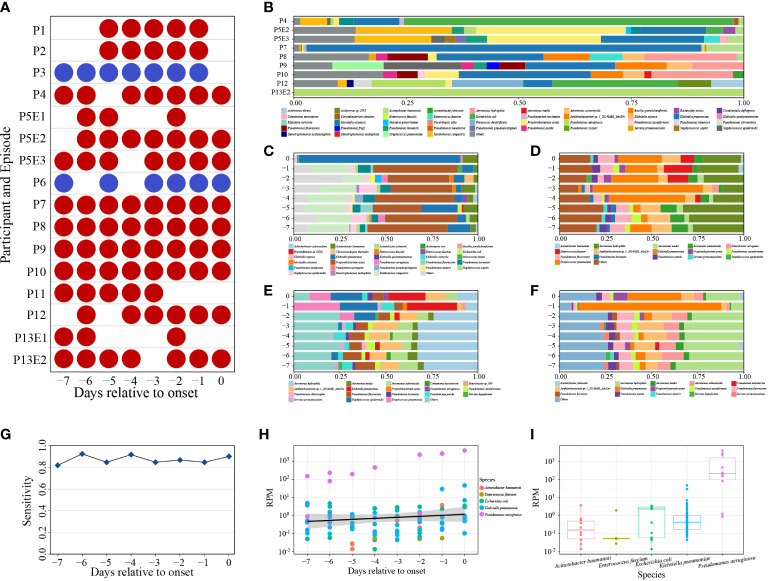
**(A)** Positive (red) and negative (blue) mcfDNA-seq results. **(B)** The percentages of the top ten bacteria of positive mcfDNA-seq samples on day of BSI onset. Species were sorted by name. **(C–F)** Variable percentages of the top ten bacteria of P7, P8, P9, P10. Species were sorted by name. **(G)** Sensitivity of mcfDNA-seq by days. **(H)** Variety of BSI pathogen-specific RPM by days. Circles represent RPM values of specific pathogens. Different colored circles represent different species. Black line indicates fitting curve of RPM. **(I)** RPM of pathogens. Circles represent RPM values of specific pathogens.

Of 100 samples subjected to mcfDNA-seq, 87 were positive (**Figure 4A**). Nine of the ten samples submitted to mcfDNA at the onset of BSI were concordant with culture results except the sample of P6. Blood culture of P6 yielded *S. hominis*, while the top five species identified by mcfDNA-seq on Day 0 were *A. baumannii*, *P. aeruginosa*, *K. pneumoniae*, *S. epidermidis* and *Corynebacterium simulans*; *S. hominis* was not identified (**Supplementary Table 1**). Before the onset of BSI, 78 sequencing samples from 14 BSIs were positive; pathogens were identified by mcfDNA-seq in 14 of 16 sequential sets of samples of BSIs. Sequential mcfDNA-seq samples taken before the onset of BSI of P3 and P6 yielded discordant results with blood culture. The sequential mcfDNA-seq samples of P3 identified mainly *A. baumannii*, *K. pneumoniae* and *E. coli*, but not *E. faecium* (**Supplementary Table 1**). Sequential mcfDNA-seq samples from P6 including a sample taken at the onset of BSI identified mainly *K. pneumoniae*, *A. baumannii*, *Xanthomonas campestris*, but not *S. hominis* (**Supplementary Table 1**).

At the onset of BSI, we detected five cultured pathogens in nine sequenced samples, the proportion of which ranked highest in P4, P7, P8, P9, P10 and P13E2 and ranked second in P5E3 and P12 (**Supplementary Table 1**). Pathogenic bacteria accounted for the majority of the top ten bacteria (**Figure 4B**). Samples of P7, P8, P9 and P10 were collected daily and underwent sequential mcfDNA-seq. The composition and percentage of the top ten bacteria fluctuated between days (**Figures 4C–F**), respectively. Time-series analysis showed an increased proportion of *K. pneumoniae* identification within the three days prior to the onset of BSI. However, the percentage of *K. pneumoniae* identification during the early samples varied greatly.

The diagnostic sensitivity of mcfDNA-seq was 90% (9/10), while total predictive sensitivity was 87.5% (14/16). Daily predictive sensitivity of mcfDNA-seq ranged from 81.82% to 92.31%, which were all greater than 80% (**Figure 4G**).

RPM typically increased in the days approaching BSI onset (**Figure 4H**). However, there was a large RPM range between pathogens, and the RPM of the same bacterium also varied greatly (**Figure 4I**). *P. aeruginosa* displayed the highest RPM, ranging from 8.6×10^-1^ to 4.1×10^3^. The most frequently identified pathogen, *K. pneumoniae*, exhibited an RPM range from 4.3×10^-2^ to 4.6×10^1^. The RPM of the other three pathogens ranged from 1.3×10^-2^ to 3.7×10^0^.

## 4 Discussion

Metagenomic next-generation sequencing (mNGS) has received increasing attention as a clinical tool for pathogen detection. Multiple reports have demonstrated the advantages of mNGS to identify etiologic agents in diverse infections (Click et al., 2018; Hong et al., 2018; Fernandez-Carballo et al., 2019; Langelier et al., 2020; Chen et al., 2021). In addition, plasma mcfDNA may be an indicator of the onset of sepsis (Martins et al., 2000; Rhodes et al., 2006; Grumaz et al., 2016). Furthermore, mcfDNA-seq can not only be used for etiologic diagnosis, but may potentially predict BSI and thereby inform preemptive antimicrobial treatment.

The high diagnostic sensitivity and predictive value observed in our study suggests that mcfDNA-seq can facilitate BSI diagnosis and predict pathogens before BSI develops. The percentage of pathogenic bacteria was relatively high both in diagnostic and predictive samples. Furthermore, the RPMs of specific pathogens trended upward during the days approaching BSI onset, which could be related to the increasing microbial load in samples, as positive blood culture requires a significant inoculum of circulating bacteria. Blood culture requires 40-80 mL blood, which is a great burden on the participants. In the early stages of bacteremia, blood cultures may be negative. McfDNA-seq may have served as a “liquid biopsy” to diagnose occult focal infections before the onset of bacteremia, or may have detected early bacteremia below the threshold of detection of blood cultures, and requires much less blood than culture. Unfortunately, six participants died of BSI in our study. Thus, it is necessary to develop new pathogen detection methods to enable pre-emptive antibiotic therapy.

Despite these advantages of mcfDNA-seq, some limitations must be noted. In two of our participants, mcfDNA-seq did not detect pathogens identified by blood culture. This finding might have several explanations. First, the low microbial load in early samples requires deeper sequencing, and the sensitivity of mcfDNA-seq is highly dependent on the depth of sequencing. Second, mcfDNA is a remnant of microorganisms killed by anti-infective drugs and immune response (Gutierrez et al., 2019; Han et al., 2020) and has a very short half-life (Elshimali et al., 2013; Grumaz et al., 2016). The low content of mcfDNA or degraded mcfDNA further increased the difficulty of pathogen detection. In addition, mcfDNA analysis may be not suitable to detect intracellular pathogens (Chiu and Miller, 2019); consequently, the real-world clinical impact of plasma mNGS is still controversial (Hogan et al., 2021). In our study, it would be better to sample directly from participants instead of using residual blood samples. And our work lacked healthy volunteer’s specimens. The specificity of mcfDNA-seq remains to be further evaluated. Prospective clinical studies in larger cohorts including clinicians and healthy subjects are necessary to further evaluate mcfDNA as a tool for early or pre-emptive diagnosis.

Above all, our study demonstrated that mcfDNA-seq, a hypothesis-free diagnostic approach, may identify and predict pathogens causing BSI. Our findings may further assist the determination of the possible clinical utility of mcfDNA-seq.

## Data availability statement

The data presented in the study are deposited in the China National GeneBank DataBase (CNGBdb) repository, accession number CNP0004130.

## Ethics statement

The studies involving human participants were reviewed and approved by the ethics committee of the sixth medical center of PLAGH. Written informed consent for participation was not required for this study in accordance with the national legislation and the institutional requirements.

## Author contributions

YL, PL, HS and YC conceived and designed the experiments. YC, XF and PD collected relative samples. TJ, YL performed the experiments. YC, TJ, YL, and HS participated in data analysis. YC, TJ and YL contributed reagents and materials. YC and PL wrote and revised the manuscript. All authors agree to be accountable for the content of the work. All authors contributed to the article and approved the submitted version.
